# Optimizing Effective Parameters to Enhance the Sensitivity of Vertical Flow Assay for Detection of *Escherichia coli*

**DOI:** 10.3390/bios12020063

**Published:** 2022-01-25

**Authors:** Manpreet Kaur, Evgeni Eltzov

**Affiliations:** 1 Department of Postharvest Science, Institute of Postharvest and Food Sciences, The Volcani Institute, Agricultural Research Organization, Bet Dagan 50250, Israel; manpreet.kaur@mail.huji.ac.il; 2 Institute of Biochemistry, Food Science and Nutrition, Faculty of Agriculture, Food and Environment, The Hebrew University of Jerusalem, Rehovot 76100, Israel; 3 Agro-Nanotechnology and Advanced Materials Research Center, Institute of Postharvest and Food Science, Department of Postharvest Science, Agricultural Research Organization, The Volcani Institute, Rishon LeZion 7505101, Israel

**Keywords:** vertical flow, immunoassay, colorimetric, lactose, polyvinyl alcohol, sensitivity

## Abstract

Vertical flow immunoassays (VFIAs) are considered potential point-of-care testing (POCT) devices compared to lateral flow assays due to their ability to analyze a comparatively large sample volume and ease of multiplexing. However, VFIA devices are limited by low analytical sensitivity when coupled with a visual colorimetric signal. Herein, we carefully analyzed key parameters that accounted for the proper functionality of VFIA that can be modified to enhance the overall sensitivity of VFIA. In particular, we focused on improving the stability of conjugate pads impregnated with capture antibodies, maintaining a controlled flow rate to ensure higher analyte reactivity with capture antibodies, and enhancing the absorption efficiency. The results showed that air-drying of conjugate pads in the presence of 5% (*w*/*v*) lactose significantly improved the stability of antibodies during long-term storage. Integration of dissolvable polyvinyl alcohol (PVA) membrane of optimal concentration as a time-barrier film into the sensor delayed the flow of samples, thereby increasing the biorecognition interaction time between immunoreagents for the formation of immuno-complexes, which in turn led to higher sensitivity of the assay. Furthermore, the employment of an absorbent pad with higher water holding capacity significantly reduced the non-specific binding of immunocomplexes, thereby reducing the possibility of false-negative results.

## 1. Introduction

In recent years, there has been an increasing demand for point-of-care (POC) multiple diagnostic assays that allow rapid detection of analytes present in samples compared to conventional lab-based testing. A wide range of biosensors based on lateral flow immunoassay (LFIA) is employed in the healthcare sector, environmental sector, and food industries [1,2,3]. LFIA is based on affinity interactions such as hybridization of aptamer-target, antibody-antigen, biotin-streptavidin, or probe DNA-target DNA hybridization and offers advantages over conventional analytical methods in terms of low cost, on-site response, ease of operation, portability, robustness, and fast and naked-eye detection [4]. On the other hand, the LFIA approach also has limitations, including its relatively low sensitivity and sample volume constraints [5]. To overcome the restrictions typically associated with LFIAs, a vertical flow immunoassay (VFIA) design is deemed a potential alternative. VFIAs are reported to provide the benefits of enhanced sensitivity, owing to the use of larger sample volumes, increased speed, and the absence of the ‘hook-effect’ [5,6,7]. VFIA comprises similar materials employed in LFIA; however, the membranes are stacked, and the sample applied vertically migrates from the upper layer to the bottom layer. Various VFIAs have been developed so far to detect antibodies, biomarkers, and biothreat agents [5,6,8,9,10].

**Figure 1:**
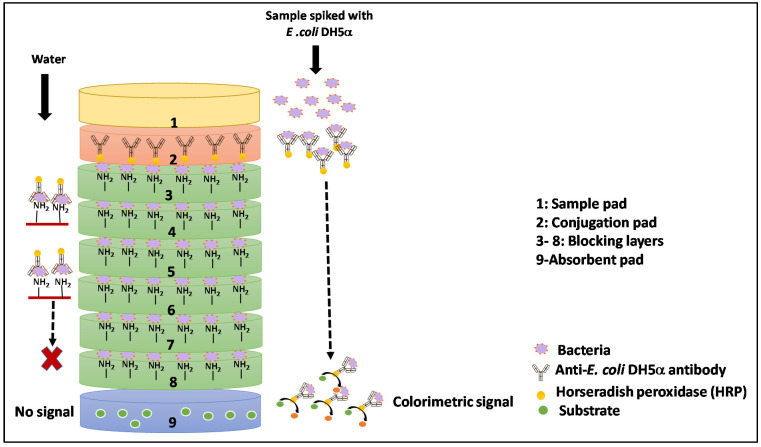
Schematic description of the vertical flow immunoassay and the assessment process. As the spiked sample is added to the sample pad, the bacteria bind to antibodies on the conjugate pad and form antibody-bacterial complexes that eventually migrate through the pads to the bottom-placed absorbent pad, thereby generating a positive colorimetric signal. In negative control without bacteria (water), free antibodies get released from the conjugation pad along with sample medium and bind to the immobilized *E. coli* cells on the blocking layers and thus do not proceed to the absorbent pad, producing no signal.

One such VFIA was developed in our previous study to detect *Escherichia coli (E. coli)* DH5α in milk samples to monitor milk quality and safety [11]. It employed a multiple-membrane set-up comprising (from top to bottom): a sample pad, a conjugation pad (with anti-*E. coli* antibodies conjugated to horseradish peroxidase), six blocking membranes with immobilized *E. coli* DH5α, and an absorbent pad with the dried substrate (TMB (3,3’,5,5’-Tetramethylbenzidine)) (Figure 1). After addition, the sample diffuses from the sample pad to the conjugation pad, where the bacteria present in the milk sample conjugates with anti-analyte antibodies conjugated to horseradish peroxidase (HRP). The resultant antibody-*E. coli* complex then migrates to the blocking layers with the immobilized analyte (*E. coli* DH5α), which selectively binds to and stops the migration of free/unbound antibodies while allowing the antibody-*E. coli* complex to pass through the blocking layers to the absorption pad, indicating positive colorimetric detection. As this approach enables the rapid detection of bacteria in a cost-effective manner, it could be employed as a portable POC testing (POCT) tool to identify pathogenic bacteria in milk for which an antibody is available.

However, despite the advantages of this VFIA set-up, a few restraints that limit its potential as POCT includes instability of capture antibodies and inefficient release of antibodies from the conjugate pad. Furthermore, the fast flow rate of the sample through the stacked membranes provides less reaction time for the analyte to bind (*E. coli* DH5α) to capture the antibody, thereby increasing the odds of false-negative results. Hence, the present study attempts to optimize critical parameters to improve the overall sensitivity of VFIA. The parameters examined included the composition of antibody coating buffer, the temperature of drying the conjugate pad, the inclusion of a time-barrier layer to delay sample flow rate, and the use of an absorption pad with better fluid holding capacity. Herein, we demonstrated that through judicious optimization of parameters, the overall sensitivity and reproducibility of the VFIA can be increased manifold. The modifications made in the VFIA design in this study are non-specific and, hence, could be applied to any vertical flow immunoassay system to obtain a high signal intensity.

## 2. Materials and Methods

### 2.1. Reagents and Membranes

Luria broth (LB) (L3022), tween-20 (cat. No. P7949), boric acid (B6768), polyvinyl alcohol (PVA-30–70 K, 87–90% hydrolyzed (cat. No. 8136)), and (3-Glycidyloxypropyl)trimethoxysilane (GPTMS, cat. No. 440167) were purchased from Sigma Aldrich (St. Louis, MO, USA). Glacial acetic acid (cat. No. 64-19-7) was purchased from Gadot (Netanya, Israel). Methanol (MeOH, cat. No. 67-56-1) was purchased from Bio-Lab (Jerusalem, Israel). Hydrogen peroxide (H_2_O_2_ (30% (*w*/*w*)), cat. No. 7722-84-1) was purchased from Carlo Erba (5347 JW, OSS, Netherlands). Phosphate buffered saline (PBS) tablets (cat. No. P4417) and sodium periodate (cat. No. 7790-28-5) were procured from Fisher chemicals (Chicago, IL, USA). PBS (0.05% *v*/*v*)–tween solution (PBST) was prepared by adding 0.5 mL Tween-20 solution to 1 L PBS (0.01 M NaH_2_PO_4_, 0.0027 M KCl and 0.137 M NaCl, pH 7.4). Borate buffer (1 M, pH 7.4) was prepared by dissolving 0.1 M of boric acid with 0.075 M of NaOH. Hydrochloric acid (HCl, (37% (*w*/*w*)), cat. No. 7647-01-0) was purchased from Chem-Lab NV (Belgium). Sucrose (cat. No. 6138-23-4), β-lactose (cat. No. 5965-66-2) and trehalose (cat. No. 6138-23-4), skim milk powder (cat. No. 70166) and 3,3′,5,5-tetramethylbenzidine (TMB) (cat. No. T0440) were purchased from Merck (Darmstadt, Germany). Skim milk solution (5% *w*/*v*) was prepared by adding 5 g skim milk powder to 100 mL PBST. Milli-Q ultrafiltered H_2_O (Milli-Q, Millipore Co., Bedford, MA, USA) was used to prepare all solutions. The pads include sample (cat. No. GFB-R4), absorbent (cat. No. AP-080, AP-0110, and AP-0120), and conjugate-release (cat. No. PT-R5) pads, purchased from Advanced Microdevices Pvt. Ltd. (Ambala, India). Anti-*E. coli* DH5α antibody conjugated with HRP (bs-2033R-HRP) was purchased from Bioss antibody (Woburn, MA, USA).

### 2.2. Bacterial Growth

*E. coli* (DH5α) strain was obtained from Robert Marks (Ben-Gurion University, Beer-Sheva, Israel). For each experiment, bacteria were cultured in 10 mL fresh LB and incubated overnight at 37 °C in a rotary thermoshaker MaxQ 4450 (Thermo scientific (401 Mill Creek Rd, Marietta, OH 45750, USA)) at 120 rpm. The culture was then diluted with LB and regrown at 26 °C to the early log phase of 10^7^ cells/mL (optical density at 600 nm (OD_600_) = 0.2) as determined by an ultrospec 2100 Pro spectrophotometer (Amersham, Cambridge, UK). Subsequently, 10 mL culture was pelleted by centrifuging at 7840× *g* for 5 min and washed with double distilled water. This step was repeated three times. The bacteria were then diluted (1:2000) in PBS to the final test concentrations (5 × 10^3^ cells/mL).

### 2.3. Preparation of Activated Membranes

The polyester membrane was immobilized with *E. coli* using the silanization method [12]. The polyester membrane pads were first to cut into 1 cm ×1 cm strips using a paper cutter. To clean the fibers from microscopic particles, the pads were dipped in MeOH: 37% HCl solution (1:1) for 20 min, after which they were thoroughly washed with double distilled water (ddH_2_O). The cleaned membrane pads were activated in piranha solution (H_2_O_2_: HCl, 7:3 *v*/*v*) for 10 min at 90 °C. Piranha solution improves the exposure of hydroxyl groups (OH) on the silica surface so that silane can attach to them by a covalent bond. After activation, the membrane pads were washed thoroughly in ddH_2_O and dried in an oven (120 °C) for 60 min. The activated and dried membranes were then immersed in GPTMS (98%, *v*/*v*) in glass tubes at 60 °C for 60 min. After that, the silanized membrane surfaces were exposed to 11.6 mM HCl for 60 min to encourage the formation of vicinal diols. After washing with ddH_2_O, the membrane pads were dipped in 100 mM sodium periodate (NAIO_4_) dissolved in 10% (*v*/*v*) acetic acid at room temperature (RT) in the dark for 60 min. This step is based on the Malaprade reaction that oxidizes the vicinal diols to terminal aldehyde groups. The pads were again rinsed with ddH_2_O and employed to immobilize *E. coli* (DH5α), as mentioned in Section 2.4.

### 2.4. Preparation of Blocking Layers

Blocking layers were prepared by incubating the activated membranes with *E. coli* DH5α solution (5 × 10^3^ cells/mL) overnight at 4 °C on an orbital shaker (120 rpm). These membranes were then washed three times with PBST to remove unbound bacteria and incubated with blocking solution (5% (*w*/*v*) skim milk and 0.05% (*v*/*v*) Tween 20 in PBS) for 1 h. Finally, the membranes were washed three times with PBST and dried at 30 °C. The blocking layers were then prepared by punching 6 mm diameter circles from the blocked membrane strips.

### 2.5. Preparation of PVA Films

The PVA films of varying concentrations (5%, 7%, 10%, 15% (*w*/*v*)) were made using the drop-casting method. The PVA was first weighed and then added into deionized water according to the required concentration. Once added, the solution was placed on a hot plate (80 °C) and stirred at 250 rpm for 1 h to obtain a homogenous solution. Then, 10 mL of the homogenous PVA solution was placed in a disposable plastic Petri dish (Miniplast, Israel), and the pates were kept in the oven at 37 °C for 22 h.

### 2.6. 3D-Printing of the Customized Holder

The customized 3D-printed holder was modeled using Autodesk Fusion 360 software and printed using MakerBot 3D Printer Method X (Stratasys, Rehovot, Israel) with polylactic acid (PLA) and polyvinyl-alcohol (PVA) as supporting material. Once printed, the prototype was washed using Ecoworks^TM^ Cleaning Agent (Eden Prairie, MN, USA) to remove the excess support material. All aqueous solutions were prepared using Milli-Q ultrafiltered (UF) deionized water.

### 2.7. Assembly of the Vertical Immunoassay System

The system was assembled by stacking all prepared membranes, one on top of the other, in the order (from top to bottom): sample pad, conjugation pad, six blocking layers, and absorbent pad (Figure 1). To provide optimal time for the occurrence of immunoreactions and allow a one-directional flow of sample, the entire setup was placed in a 3-D printed plastic holder (Figure S1). 

### 2.8. Instrumentation

The presence of HRP-conjugated immunocomplex in the absorbent pad was detected using the sensitive chemiluminescence reaction. After adding the sample, all the layers of VFIA set-up were placed in a 96-well plate. To prevent the differences between reaction processes on the different membranes, the substrate solution (luminol: H_2_O_2_ (1:1, *v*/*v*), cat. No. 1705040, BioRad, Rehovot, Israel) was added simultaneously using a multichannel pipette and read at the same time. The plate was run kinetically for 10 min at an interval of 2 min with 6 reads per well. The signal at which the enzymatic reaction came to the steady-state phase was taken as the final relative light unit (RLU) value.

The chemiluminescence was analyzed by reading the plate in a plate reader (BioTek EL808-I Ultra Microplate Reader (BioTek Instruments Inc., Winooski, VT, USA).

### 2.9. Optimization Steps

#### 2.9.1. Optimization of Antibody Stability in Conjugation Pad

Conjugation membranes were prepared by punching 6 mm diameter circles from the polyester membrane using a paper puncher. Pre-treatment of conjugate pads is crucial to ensure the stability of antibodies. The effect of the chemical and physical parameters on the immobilized antibodies’ stability and functionality was optimized using different buffers at different temperatures. 

Buffers for blocking of conjugate pads were prepared as described:

(a) Borate buffer (5 mM, pH 7.4) containing 0.05% (*v*/*v*) Tween 20
(b) PBS (10 mM, pH 7.4) buffer containing 0.05% (*v*/*v*) Tween 20
(c) PBS (10 mM, pH 7.4) containing 0.05% (*v*/*v*) Tween 20 with 5%, 10% or 20% (*w*/*v*) sucrose
(d) PBS (10 mM, pH 7.4) containing 0.05% (*v*/*v*) Tween 20 with 5%, 10% or 20% (*w*/*v*) lactose

The antibodies were first diluted (1:1000) in different blocking buffers, and conjugate pads were immobilized by applying 20 µL of antibodies solution using a micropipette. The conjugate pads were dried under two other conditions, viz., at 37 °C for 60 min and in a fume hood under an airflow rate of 0.75 m/s for 90 min. The dried conjugate pads were stored under different temperatures viz., at room temperature (RT), 4 °C, and −20 °C for 24, 48, and 72 h, to determine the most effective method to ensure the stability of antibodies during long term storage. The antibody functionality was assessed every 24 h by measuring the luminescence exhibited by conjugate pads in a 96-well plate in a plate reader.

#### 2.9.2. Optimization of Conjugate Release

The pre-treatment of conjugate pads is also critical to ensure the consistent and uniform release of antibodies upon hydration. It has been reported that the addition of carbohydrates in pre-treatment buffer facilitates the release of conjugate from the conjugate pad. Hence, to select the disaccharide that enhances the antibody release, in this study, conjugate pads were immobilized with antibodies diluted in 10 mM PBST buffer containing varying concentrations (5%, 10%, and 20% (*w*/*v*)) of different sugars (sucrose, lactose, and trehalose). The sugars were selected based on their observed effect on antibody stability during long-term storage, as explained in Section 2.9.1. After drying, the antibody-coated conjugate pads were placed in the 3D-printed holders in the stacked-pad arrangement of membranes (from top to bottom)-sample pad, conjugate release pad, and six clear polyester membranes (without immobilized *E. coli* DH5α) and absorbent pad. The PBS buffer (90 µL) was added gradually to allow the antibodies to migrate towards the absorbent pad. The membranes removed from the 3D-holders were added with luminol: H_2_O_2_ (1:1, *v*/*v*) solution and luminescence was measured in a 96-well plate in the plate reader.

#### 2.9.3. Optimization of Flow Management 

Maintaining a controlled sample flow rate in the designed VFIA is necessary for forming the antibody-analyte complex. Hence, to impede the antibodies released from the conjugate pad upon wetting, PVA films of varying concentrations (5%, 7%, 10%, 15% (*w*/*v*)) were placed beneath the conjugate pad to allow the binding of antibodies to the analyte (*E. coli* DH5α) present in the sample. PVA films were prepared by punching 6 mm diameter circles from the synthesized PVA sheet and were integrated beneath the conjugation pad into the customized 3D-printed holders. A separation pad was placed between the conjugation pad and PVA film to minimize the direct contact of antibodies with dissolved PVA. After the PVA films were integrated into the holder, a colored dye was then added through the top entrance of the holder. The dissolution times of tested films were measured after observing the visible flow of the ink sample into the absorbent pad. Furthermore, to determine whether the integration of a PVA layer in VFIA could negatively affect the activity of the antibody/HRP enzyme, anti-*E. coli* DH5α antibody at different dilutions was incubated with studied concentrations of PVA solutions in a 96-well plate, and antibody/HRP functionality was assessed by measuring the chemiluminescence intensity. 

#### 2.9.4. Optimization of Absorption Efficacy

The absorbent pad is a critical component of VFIA as it is placed at the bottom of the vertical stack-pad design to absorb the excess sample volume, preventing its backflow. Absorbent pads with high absorbing capacity could allow an analysis of a comparatively large sample volume, thereby enhancing the sensitivity of VFIA. Therefore, to determine the effect of absorption efficiency on the performance of VFIA, three types of absorbent pads, *viz*., AP080, AP0110, and AP0120, with different characteristics were employed (Table 1). The absorbent pads were placed in the VFIA set-up in a similar way as described in Section 2.7, and PBS (90 μL) was applied gradually to the membrane stack placed in the plastic holder. After that, the membranes were removed from the holder, and their luminescence was measured as described in Section 2.9.2. 

## 3. Reproducibility and Statistical Analysis 

One-way repeated-measures analysis of variance (ANOVA), followed by Tukey’s post hoc test, was employed to evaluate the dispersion between different tested parameters. The error bars represent the standard error of independent replicates and biological samples, and the marking in the figures indicates the level of statistically significant difference (“*” when *p* < 0.05 and “**” when *p* < 0.01). The data were analyzed with Graphpad Prism (Graphpad Software, Inc., Version 5, San Diego, CA, USA). 

## 4. Results and Discussion

### 4.1. Optimization of the Efficacy of Conjugate Pad

Conjugate pads play a pivotal role in regulating the performance of paper-based vertical/lateral flow assays. The function of the conjugate pad is to retain the antibodies and maintain their functional stability until the assay is conducted. This is assured by blocking the conjugate pads with a blocking solution containing buffer salts and sugars (sucrose, trehalose), which function as a preserving and rehydrating agent. 

#### 4.1.1. Selection of Base Buffer 

The base buffer used to apply the conjugate onto the conjugate pad is a critical factor for the VFIA as buffer ions interact with antibodies, affecting their conformation and reactivity. Adding buffer salts to the conjugate pad can also minimize the variation exhibited by samples with different pH and ionic strength [13,14,15]. Hence, selecting a suitable base buffer is essential as it stabilizes the reaction elements for improved performance of VFIA. Previous reports have suggested the use of a borate buffer as a blocking reagent, owing to its superior performance for LFIAs [16,17,18]. Hence, based on the preliminary results, we compared the performance of two different base buffers, *viz.*, borate buffer (5 mM, pH 7.4) and PBS (10 mM, pH 7.4). Both the buffers were added with 0.05% (*v*/*v*) Tween-20 as it promotes re-solubilization of the conjugate, reduces nonspecific binding of the conjugate, and minimizes the analyte’s adsorption to the membrane.

**Figure 2:**
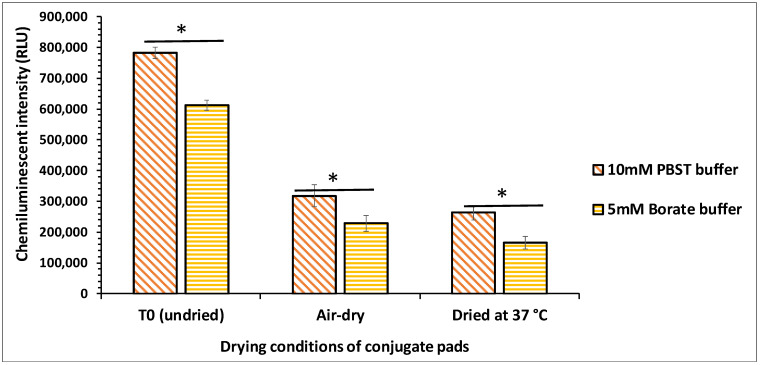
Effect of 5 mM borate and 10 mM PBST buffer on the stability of conjugate pads during the drying process (* *p* < 0.05 by ANOVA).

Among the tested buffers, 10 mM PBST (PBS with Tween-20) showed an enhanced effect on antibody stability during the drying process, as shown in Figure 2. This may be because compared to borate buffer, PBST could more efficiently form hydrogen bonds with the reporter molecule before the drying process, which enabled the maintenance of the native form of the antibodies [14]. PBS at 10 mM concentration has also been shown to improve the thermostability of the HRP enzyme by significantly increasing its half-life from 13 to 35 min at 80 °C [19]. 

#### 4.1.2. Optimization of Stabilizers and Drying Temperature of Reporter

**Figure 3:**
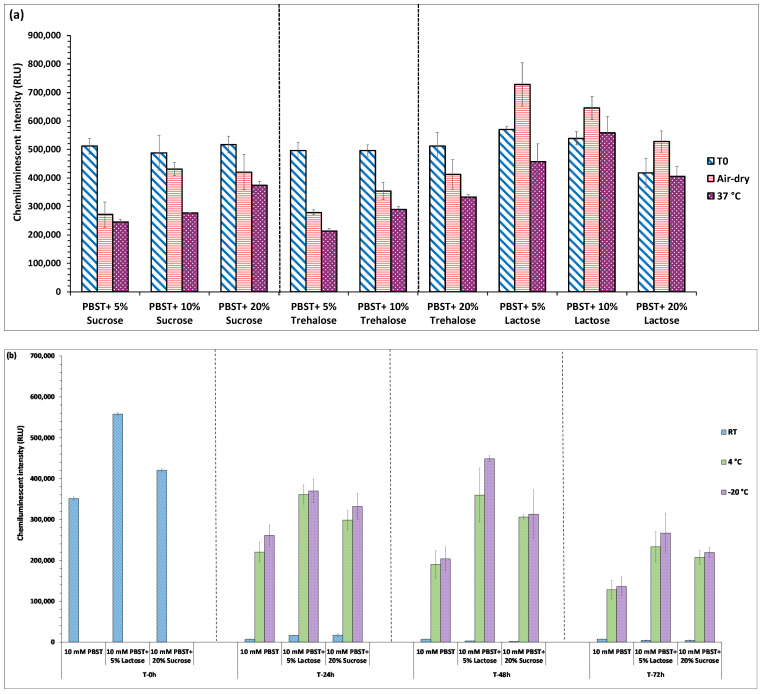
Impact of different types of stabilizers on the stability of conjugate pads during (**a**) drying process and (**b**) storage at different temperatures.

The addition of sugars in the buffer has been reported to enhance the stability of antibodies formulations. When the conjugate pad is dried in the presence of sugar, the sugar molecules form a layer around antibodies, thereby retaining their biological/structural integrity [20,21]. The sugar molecules rapidly dissolve when the sample diffuses through the conjugate pad, carrying the antibodies into the fluid stream. There are conflicting reports regarding the role of different types of sugars in maintaining protein’s stability. Various studies reported trehalose as an efficient stabilizer because of its higher glass transition temperature (Tg) as compared to sucrose [22,23,24]. On contrary, there are studies that have demonstrated the employment of sucrose for preserving protein structure [17,25,26]. Hence, in this study, to determine the effect of sugars on the integrity and thermal stability of antibodies, they were diluted in PBST buffer containing different types of sugars, viz., sucrose, trehalose, and lactose, at three different concentrations (5%, 10%, and 20% (*w*/*v*)). After applying conjugate on the pads, they were exposed to two different drying conditions, viz., at 37 °C for 60 min and air-dry at RT for 90 min. The efficacy of dried pads impregnated with sugar-supplemented conjugate was compared to the undried pads added with the conjugate just before chemiluminescent analysis (T0). The results showed that 5% (*w*/*v*) lactose significantly improved the stability of dried conjugate pads among tested sugars (Figure 3a). This is probably because lactose interacts stronger with biomolecules than sucrose and is more effective in protecting proteins. Lactose also exhibits low hygroscopicity, excellent compatibility with active ingredients, and remarkable physical and chemical stability [27]. Interestingly, the dried pads impregnated with 5% (*w*/*v*) lactose exhibited higher signal strength than undried ones (T0). This could be because of less aggregation due to the homogenous dispersion during drying, leading to high adsorption of the lactose-laden conjugate on the conjugate pads. However, with a further increase in the concentration of lactose, a drop in chemiluminescent signal intensity was observed. This is probably due to the lower solubility of lactose at high concentrations. Supplementation of PBST buffer with sucrose also exhibited a protective effect on the antibodies at all the tested concentrations, as reported in various studies [15,17,18]; however, its impact was weaker than lactose. At the same time, trehalose showed minimal effect on retaining the functional integrity of antibodies at all tested concentrations. It has also been reported that trehalose is unsuitable for long-term storage at low temperatures. It is more prone to crystallization during freeze-concentration, leading to loss of cryoprotective function, thereby causing protein instability [25,26,28]. 

The drying of conjugate pads is essential to achieve long-term stability, as the presence of water facilitates the chemical and physical degradation of proteins. The drying temperature and time also significantly affect the stability of the antibodies, as subjecting the antibodies to heat stress during the drying process leads to irreversible denaturation of antibodies [26,29]. Hence, to select the optimal drying temperature, in this study, conjugate pads impregnated with antibodies were dried under two conditions viz., first set was placed in an incubator at 37 °C for 60 min and the second set was air-dried in a chemical hood at an airflow rate of 0.75 m/s for 90 min.

As shown in Figure 3a, air-drying of conjugate pads at RT preserved the functionality of the antibodies. However, conjugate pads dried at 37 °C exhibited a significant activity loss. These results revealed that air-drying of conjugate pads at a lower temperature for a comparatively longer duration is a more appropriate method as it causes minimal damage to antibodies. Although many previous studies have demonstrated drying of conjugate pads at higher temperatures (37–42 °C) for varying durations of time [17,30,31], the results of the present study clearly reveal that heat-induced stress can cause substantial denaturation of the antibody, thereby decreasing the sensitivity of VFIA.

The efficiency of the air-drying process was also demonstrated by measuring the weight of conjugate pads before applying buffer (W0) and after drying it for 90 min (W1). The conjugate pads applied with solutions devoid of sugars (PBST and water) were kept as controls. Results showed that in negative controls, the weights of conjugate pads before (W0) and after drying (W1) were similar, which implies that the drying process was efficient as it resulted in complete removal of moisture (Figure S2). However, the increased weight of conjugate pads impregnated with sugar-laden buffers after the drying process could be attributed to the weight of specific sugars as the pads applied with 20% (*w*/*v*) sucrose showed more increase in weight followed by pads loaded with 10% (*w*/*v*) lactose and 5% (*w*/*v*) lactose. Furthermore, to confirm the efficiency of the drying process, the pads were air-dried overnight under similar drying conditions. Results showed that after overnight air-drying, the weights of all pads (W2) were similar to the weight observed after 90 min drying (W1) (Figure S2), which confirms that air-drying of membrane pads for 90 min is sufficient to ensure complete moisture loss.

The use of sugars for the long-term storage of proteins is pretty common as many commercially available antibody formulations contain different sugars [22,32]. Hence, based on the stabilization efficiency of tested sugars, two best disaccharides (5% (*w*/*v*) lactose, 20% (*w*/*v*) sucrose) were selected to determine their effect on the preservation of antibodies during long-term storage at three different temperatures, viz., room temperature (RT), 4 °C and −20 °C for 72 h. Chemiluminescence analysis showed that blocking conjugate pads with sugars-supplemented PBST buffer significantly enhanced their stability compared to the pads blocked with only PBST buffer without disaccharides. The signal appeared to decrease gradually with increased duration of storage; however, the pads blocked with PBST buffer supplemented with 5% (*w*/*v*) lactose exhibited more stability both at 4 °C and −20 °C as compared to PBST buffer containing 20% (*w*/*v*) sucrose (Figure 3b). Among the tested storage temperatures, −20 °C appeared to be the optimum temperature for storing conjugate pads; however, both the sugars exhibited incompetence in preserving the stability of conjugate pads at room temperature for all-time intervals. This is attributed to the degradation or unfolding of antibodies at RT, leading to their inactivity. Therefore, owing to the sensitivity of antibodies to high temperatures (>4 °C, −20 °C), the designed VFIA device could be effectively used only at settings with lower temperature storage facilities.

### 4.2. Optimization of Conjugate Release

#### 4.2.1. Influence of Conjugate Pad Blocking on Conjugate Release

A consistent and uniform release of the conjugate is essential for VFIA to allow a smooth and fast flow of immunocomplexes towards the absorbent pad, leading to less non-specific binding and higher sensitivity. To achieve optimal antibody release, all conjugate pads were prepared by diluting the antibodies in the PBST buffer added with previously determined amounts of sugars (5% (*w*/*v*) lactose, 20% (*w*/*v*) sucrose). The conjugate pads were placed in the membrane stack in a plastic holder. As this optimization was performed to evaluate the effect of sugars on conjugate release, hence clear polyester membrane pads (without immobilized *E. coli*) were placed beneath the conjugate pads, and PBS (90 μL) was applied on top of the stack pad to facilitate the release of conjugate across the membranes.

**Figure 4:**
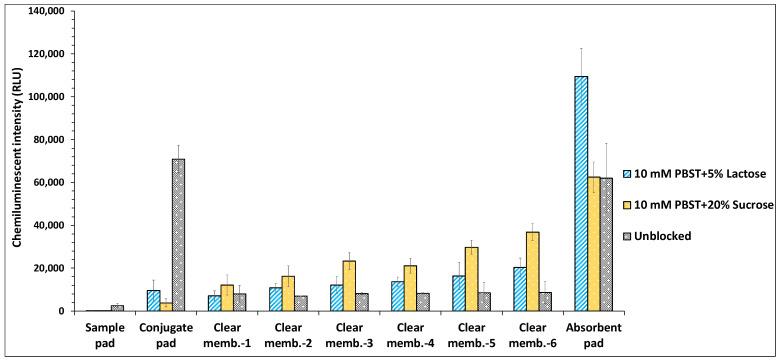
Effect of conjugate pad blocking on the release of capture antibodies.

The results showed that both the sugars (5% (*w*/*v*) lactose, 20% (*w*/*v*) sucrose) significantly enhanced the release of antibodies through the blocked conjugate pads compared to the unblocked ones coated with antibodies diluted in only PBST buffer. Among both, the disaccharides, conjugate pads treated with 20% (*w*/*v*) sucrose exhibited more efficient diffusion of antibodies as compared to pads coated with 5% (*w*/*v*) lactose (Figure 4). These results are consistent with a previous study that reported optimal antibody release at a sugar concentration of 20% (*w*/*v*) [33]. However, comparatively higher non-specific binding of released antibodies to the bottom-placed clear polyester membranes was observed for conjugate pads coated with 20% (*w*/*v*) sucrose, which led to the low signal intensity of the absorbent pad. This was probably because the high concentration of sugar (20% (*w*/*v*) sucrose) increases the viscosity of the flowing sample stream, which reduces the flow velocity, thereby increasing the interaction time between diffused antibodies and clear membrane pads [34]. On the contrary, conjugate pads treated with 5% (*w*/*v*) lactose showed minimal non-specific binding of antibodies to the membranes, which is also correlated with the observed high signal intensity exhibited by the absorbent pad. However, in an unblocked pad impregnated with buffer devoid of sugars, less conjugate transfer throughout the vertical flow set-up could be due to the binding of antibodies to the conjugate membrane surface, which hinders their release. It has been reported that blocking the conjugate pad prevents the interaction of antibodies with the membrane surface, improving the signal-to-noise ratio and facilitating the release of antibodies [33]. The other possible reason could be related to membrane fouling that might have been caused owing to the high tendency of dibasic sodium phosphate to crystallize, leading to the aggregation of antibodies. Besides eliciting the aggregation, the freezing and thawing of biotherapeutics in the presence of PBS buffer lacking stabilizers (sugars, polyols) also results in their degradation/denaturation [35,36]. Although absorbent pad of the stack pad comprising conjugate pad blocked with 5% (*w*/*v*) lactose exhibited higher chemiluminescent intensity as compared to other tested stack pads (Figure 4), however, when the intensities of all layers of each stack pad were combined, no significant difference in the integrated chemiluminescent intensity was observed for stack pads including conjugated pads blocked with 5% (*w*/*v*) lactose or 20% (*w*/*v*) sucrose (Figure S3). 

#### 4.2.2. Sample Deposition Mode

**Figure 5:**
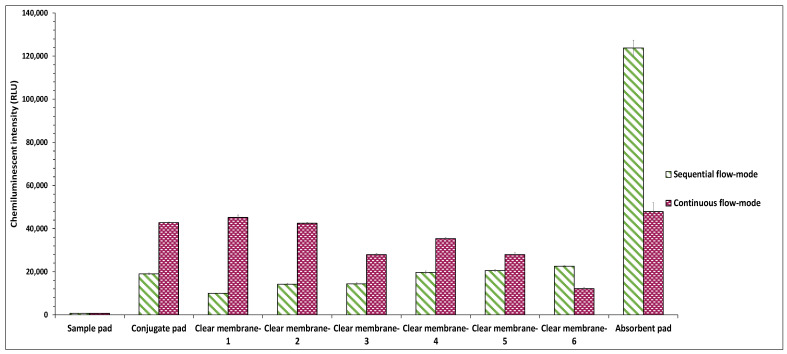
Effect of different types of sample addition modes on the conjugate release.

The method of sample addition through the vertically stacked membranes also impacts the sensitivity of the vertical-flow assay. This study evaluated the effect of two different sample deposition modes, viz., continuous-flow and sequential flow modes, on the conjugate release through the vertical flow setup. To compare the impact of the sample deposition modes on the conjugate release and sample flow pattern, clear polyester membrane pads without immobilized *E. coli* were stacked beneath the conjugate pads as described in Section 4.2.1. In the continuous-flow mode, a volume of 90 μL PBS was added through the vertical membrane stack-pad set-up in a single step, while in the sequential flow mode, 30 μL of PBS was sequentially dispensed three times at an interval of 20 s. The results showed that the sequential flow method maximized the sensitivity by causing an enhanced release of antibodies through the conjugate pad. It also prevented the non-specific binding of antibodies to the clear membranes, thereby causing the flow-through of the entire sample to the absorbent pad. In contrast, the minimal release of antibodies through the conjugate pad was noticed in continuous-flow mode (Figure 5). Furthermore, the rapid flow rate of the sample in continuous-flow mode, prevented its seepage through the membranes, thereby causing the entrapment of the sample in the membranes, limiting the sample flow to the absorbent pad. This promoted the non-specific binding of antibodies to the stacked membranes, thereby decreasing the overall sensitivity and specificity of the assay.

However, when the intensities of all layers of each stack pad were combined, the stack pad set-up, wherein the sample was added in continuous flow mode, showed higher integrated chemiluminescent intensity as compared to stack pad set-up added with the sample in sequential mode (Figure S4). This is because the observed chemiluminescent intensity of the absorbent pad in sequential mode was lower as compared to its expected value. In sequential mode, most HRP-conjugated capture antibodies directly migrated to the absorbent pad without getting trapped on the clear membranes. Owing to the fluid retention capacity of the absorbent pad, some of the capture antibodies along with the sample got entrapped in the inner layers of the absorbent pad and hence did not get exposed to the luminol substrate solution. This led to lower chemiluminescent intensity of the absorbent pad, which in turn resulted in lower integrated intensity for sequential flow mode, compared to continuous flow mode.

It is not feasible to employ the sequential sample deposition mode for LFIA owing to its different geometry; hence, this modification in VFIA gives it an advantage over LFIA in terms of sensitivity. LFIA is generally affected by mass transport limitations (MTLs) that occur as the target analyte is transported distally across the nitrocellulose membrane by passive capillary flow. Hence, the application of the sample in sequential mode could comprise the detection speed of LFIA [7].

### 4.3. Effect of PVA Layer on Flow Rate Delay 

**Figure 6:**
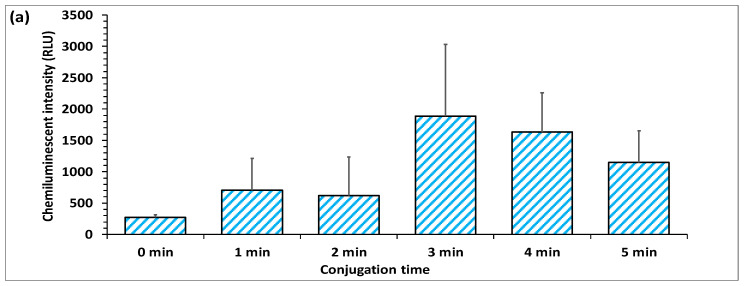

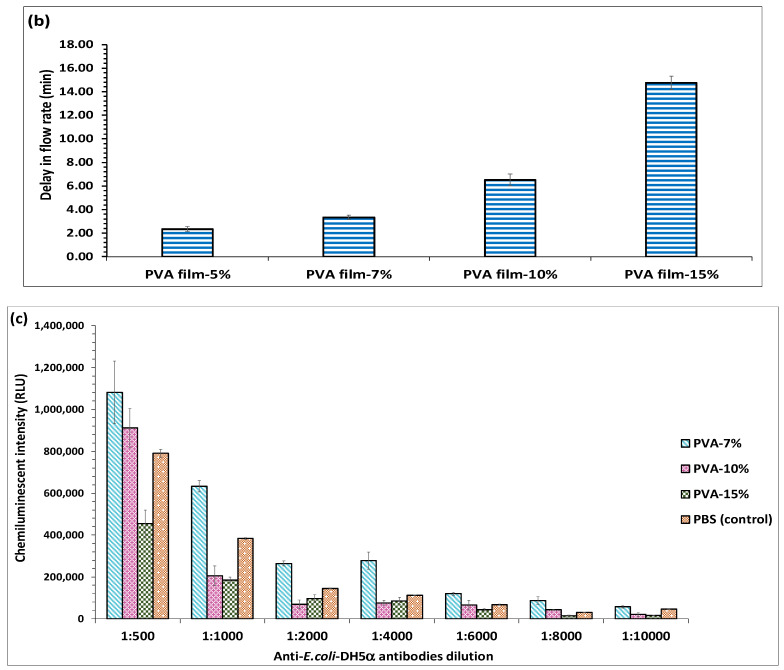
(**a**) Determination of optimal time required for the conjugation of capture antibody to *E. coli* (**b**) Effect of different concentrations of PVA films on the delay of sample flow rate (**c**) Effect of different concentrations of PVA on the activity of anti-*E. coli* antibody.

A slow flow rate in the sample reactive zone between conjugation and blocking layers is critical as it allows increased exposure time between antibodies and bacteria and leads to the formation of more antibody-bacteria complexes, which is a prerequisite for successful detection of pathogenic bacteria in the sample. This helps to ensure higher sensitivity, reactivity and reduces false-negative signals. A recent study demonstrated that the incorporation of a cellulose stacking pad in a membrane-based platform enhanced the detection sensitivity of the assay by extending the antigen-antibody binding interactions [17]. Similarly, in another study, the inclusion of polyvinyl alcohol (PVA) dam in LFIA was shown to delay the sample flow through the nitrocellulose membrane resulting in increased bio-recognition time, which in turn contributed to enhanced sensitivity of the assay [37]. Although these studies demonstrated the employment of time barrier layers made of suitable materials to increase the sensitivity of the assay, in these studies, the optimal time required to achieve maximal and effective interaction of analyte and antibody was not optimized. In the present study, we first assessed the minimal time required for the efficient coupling of *E. coli* cells and capture antibodies. The optimal time required to bind capture antibodies with bacteria was first analyzed by incubating the blocking membranes immobilized with *E. coli* DH5α with anti-*E. coli* DH5α antibodies for different time intervals (0, 1, 2, 3, 4, 5 min). The formation of antibody-bacteria complex on membranes was then determined by analyzing the chemiluminescence intensity. The results showed that the minimum conjugation time required for effective binding of the antibody to bacteria was 3 min. In comparison, the time duration of less than 3 min was incompetent as it did not allow the formation of an efficient antibody-bacteria complex (Figure 6a).

**Figure 7:**
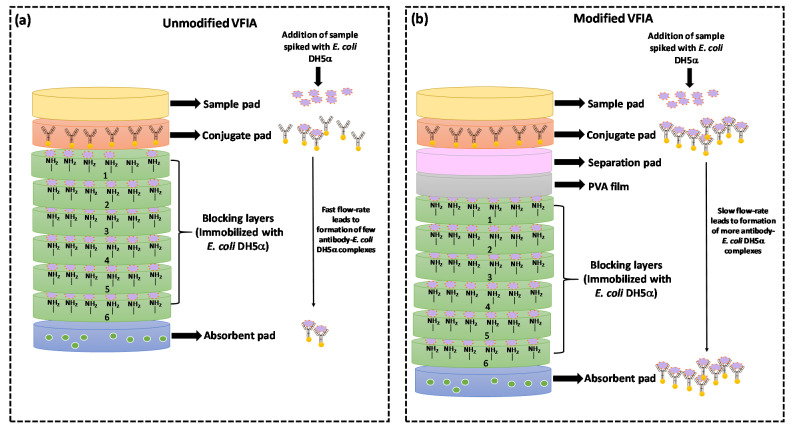
Schematic of (**a**) unmodified and (**b**) modified VFIA set-up with an integrated PVA film. For the unmodified VFIA, the fast flow rate of the sample leads to the formation of fewer antibody-*E. coli* DH5α complexes. In modified VFIA, delayed flow rate due to the presence of PVA film leads to enhanced sensitivity.

Hence, in this study, a PVA film was employed as a barrier layer inside the vertical flow set-up to obstruct the sample flow rate until the film ultimately dissolves. PVA is a water-soluble polymer as it comprises hydroxyl groups, which interact with the water molecules through hydrogen bonds [38]. A prototype to hold the PVA film in a paper-based vertical flow set-up was designed using 3D-printing technology. The PVA film was integrated beneath the conjugation pad in the customized 3D-printed holder (Figure 7), and the ability of these time-barrier films was examined by measuring the dissolution time.

PVA films of different concentrations (5%, 7%, 10%, 15% (*w*/*v*)) were prepared as the dissolution time of films is directly correlated to PVA concentration. Results showed that PVA films with lower percentages viz., 5% (*w*/*v*) and 7% (*w*/*v*) dissolved rapidly after 2 min and 3 min, respectively, making them unsuitable to be employed as a barrier layer, as they did not allow sufficient time for the binding of antibodies to bacteria. On the contrary, 15% (*w*/*v*) PVA film dissolved too slowly, taking up to 15 min, making it less effective to be used in VFIA devices. However, 10% (*w*/*v*) PVA film dissolved after 6–7 min, corresponding to the time required to form the antibody-bacterial complex as shown in Figure 6b. The results suggest that PVA films of optimum concentration can be efficiently employed in VFIA based biosensor as it effectively tunes with the antibody-analyte conjugation time [37].

The feasibility of employment of PVA layer as a barrier film in VFIA was also validated by determining its effect on the activity of antibody/HRP enzyme. To test whether the reaction of dissolved PVA with the antibodies could negatively impact the effectiveness of antibody/enzyme, different dilutions of anti-*E. coli* DH5α antibody was mixed with specific concentrations of PVA solutions that showed a delaying effect of flow rate. The antibody/HRP enzyme efficiency was assessed by measuring the luminescent signal intensity. The results showed that PVA solution at 7% and 10% (*w*/*v*) concentration did not impose any negative impact on the activity of antibody/HRP enzyme; on the contrary, it exhibited more vigorous signal intensity than the control (PBS) (Figure 6c). This is probably that at low concentration, PVA acts as a stabilizer and preserves the activity of the HRP enzyme [16,39,40,41,42]. However, with a further increase in the concentration of PVA solutions to 15% (*w*/*v*), the luminescence intensity dropped drastically. This indicates that a higher concentration of PVA inflicted a masking effect on the HRP enzyme, owing to its increased viscous nature, demonstrating its incompetency to be employed as a time-barrier film in VFIA.

### 4.4. Effect of Absorption Efficacy

**Figure 8:**
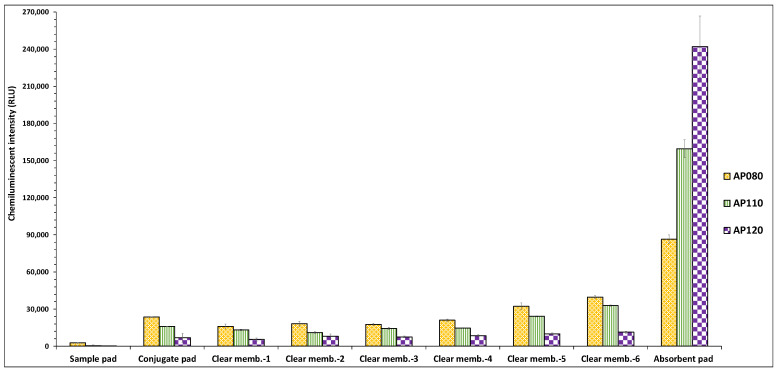
Effect of absorption efficiency of different types of absorbent pads on the sensitivity of the vertical flow assay.

The function of the absorbent pad is to increase the total volume of samples that can enter the vertical stack pad device. It is placed at the stack pad’s bottom as it acts as a sponge to absorb the additional sample running across the membrane bed [43]. This increased volume is required for the enhanced release of antibodies from the conjugate pad. An extra sample volume is also crucial to reduce non-specific binding and wash away the antibody-bacteria complex across the blocking membranes, allowing the total analyte concentration to reach the absorbent pad. This lowers the background and also assists in enhancing the assay sensitivity. The employment of AP080 absorbent pad has been demonstrated for the development of LFIA in the previously reported studies [11,44,45], while a few studies have also examined the effect of absorbent pads of varying thickness viz., AP080 and AP045 on the assay’s detection sensitivity. In one such study after preliminary optimization, AP080 was eventually selected for LFIA development owing to its increased absorption efficiency [46]. However, in another study, although no differences in the signal intensity were noticed using AP080 or AP045 absorbent pad, however, they selected AP045 for LFIA development, stating that owing to its less thickness, it allows a slower migration along the nitrocellulose membrane, which may improve the number of interactions in the control and test lines [47]. Hence, in this study, the effect of sample absorption efficiency of the absorbent pad on the sensitivity of VFIA was evaluated using three types of cellulose fiber-based absorbent pads of different physical and performance characteristics (AP080, AP0110, and AP0120). The results showed that in the membrane stack pad with the AP080 absorbent pad, the release of antibodies from the conjugate pad was minimal, and owing to the insufficient absorption capacity of the absorbent pad; the released antibodies remained entrapped in the clear membranes as indicated by higher chemiluminescent signal exhibited by all six membranes (Figure 8). However, the AP0120 absorbent pad demonstrated maximal fluid absorption followed by the AP0110 pad. It also affected the conjugate release efficiency by causing the enhanced release of antibodies from the conjugate pad. Furthermore, it also prevented the non-specific binding of antibodies on the clear membranes, thereby reducing the probability of false-negative results.

The results revealed that the absorption efficiency of the absorbent pad plays a critical role in enhancing the sensitivity of VFIA.

## 5. Conclusions

In the present study, we have reported that it is feasible to enhance the performance of VFIA by modifying critical parameters such as improving the stability of dried conjugate pads, increasing the reaction time between the analyte and capture antibody, and enabling the analysis of a large volume of samples to enhance the sensitivity of the assay. The optimal conditions that were demonstrated for preserving the conjugate pads during long-term storage included the blocking of conjugate pads with 10 mM PBST buffer containing 5% (*w*/*v*) lactose and air-drying of conjugate pads at room temperature. Furthermore, we showed that the integration of a time barrier film viz., PVA at a specific location in the sensor could pause the sample flow rate, thereby allowing the formation of an analyte-antibody complex. Enhancing the absorption efficiency by using a thicker absorbent pad significantly reduced non-specific binding, thereby improving the overall sensitivity of the assay. The modifications suggested in this study are generic, cost-effective, and could be implemented to other vertical flow immunoassays.

## Figures and Tables

**Table 1:** Physical and performance characteristics of different types of absorbent pads.

Properties	AP080	AP110	AP120
Thickness (µm)	650–950	970–1330	1120–1580
Weight (mg/cm^2^)	10–22	57–93	60–100
Water holding capacity (mg/cm^2^)	52–112	87–147	110–200

## Supplementary Materials

The following supporting information can be downloaded at: https://www.mdpi.com/article/10.3390/bios12020063/s1, Figure S1: Image of vertical flow set-up built from a series of layers; Figure S2: Depiction of the efficacy of air-drying process based on the dry-weight of conjugate pads; Figure S3: Effect of conjugate pad blocking on the integrated chemiluminescent intensity of VFIA platform; Figure S4: Effect of different types of sample addition modes on the integrated chemiluminescent intensity of VFIA platform.
