# Supplementary material for: Optimizing Effective Parameters to Enhance the Sensitivity of Vertical Flow Assay for Detection of Escherichia coli
Source: Biosensors (Basel). 2022 Jan 25;12(2):63. doi: 10.3390/bios12020063 (PMC8869093; doi:10.3390/bios12020063)
Supplement: Supplementary file 1 [file biosensors-12-00063-s001.zip › biosensors-1550770-supplementary.pdf]

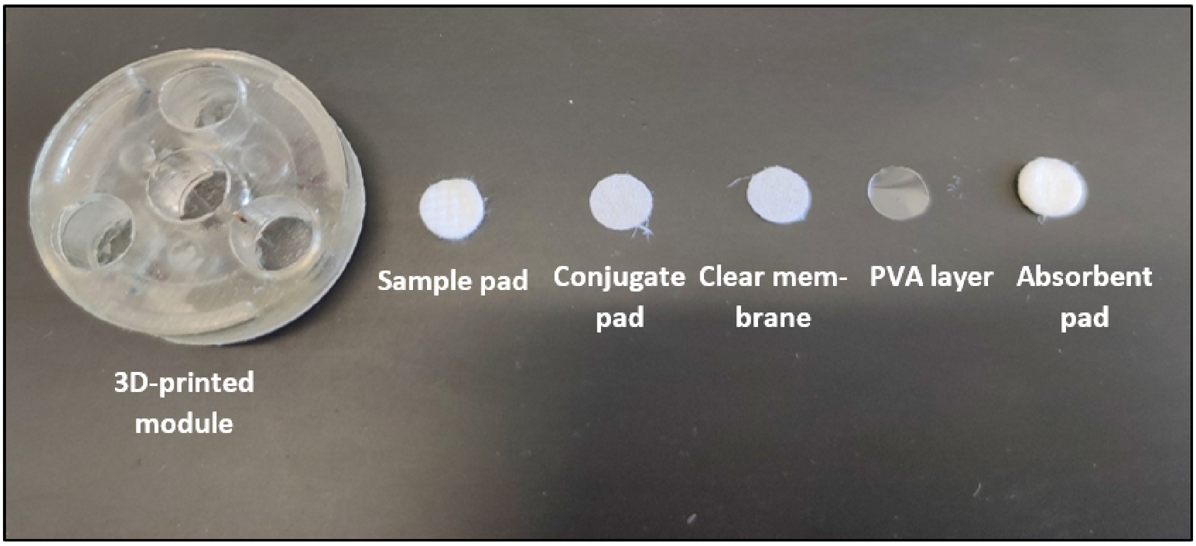

Figure S1. Image of vertical flow set-up built from a series of layers.

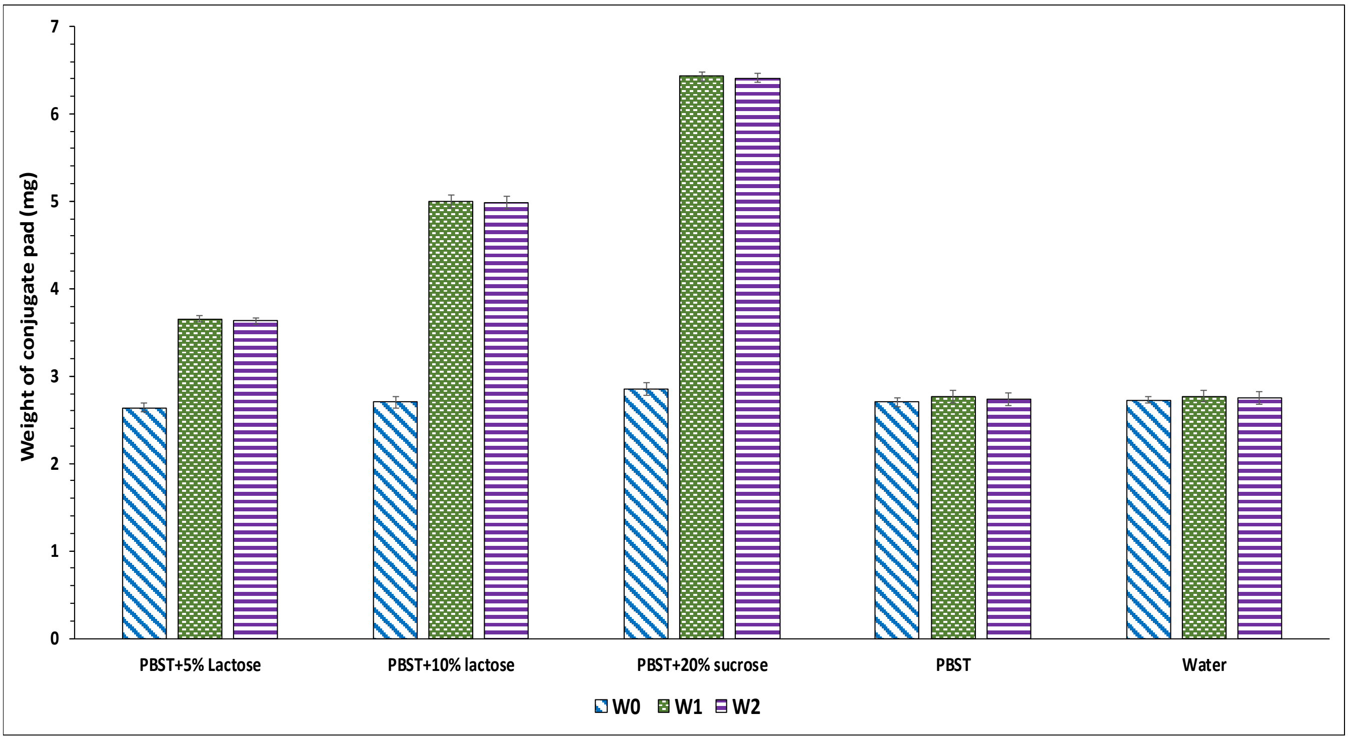

Figure S2: Depiction of the efficacy of air-drying process based on the dry-weight of conjugate pads.

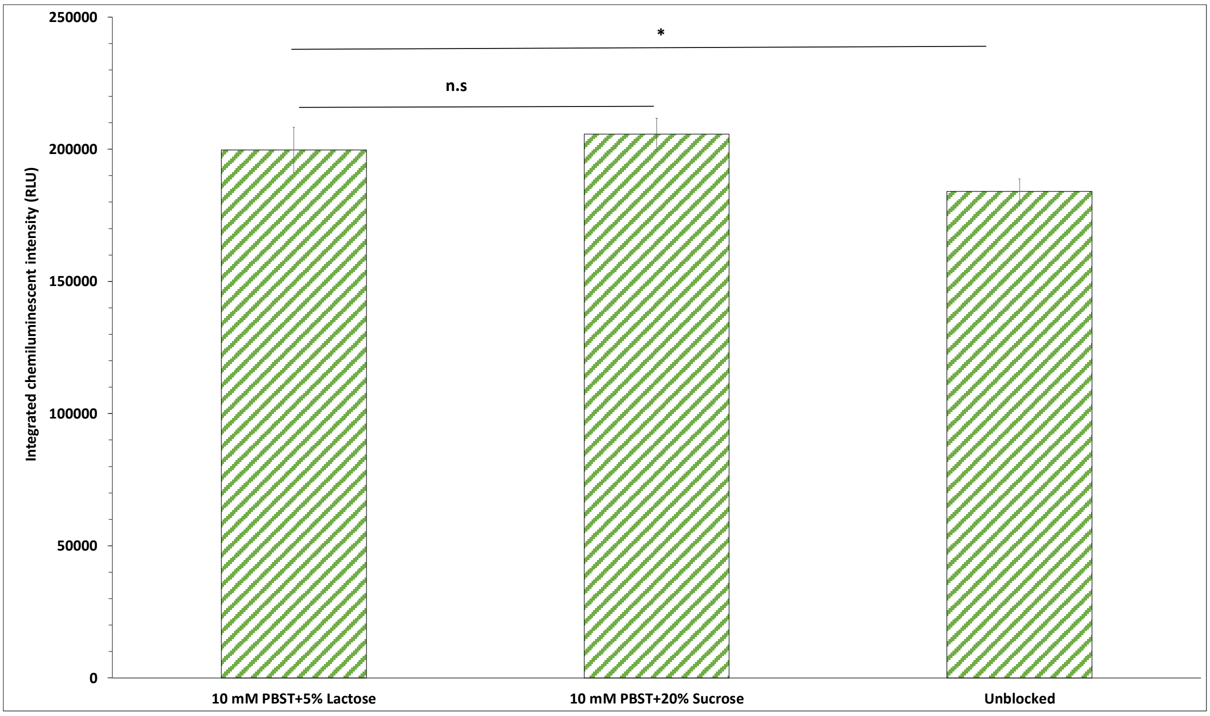

Figure S3. Effect of conjugate pad blocking on the integrated chemiluminescent intensity of VFIA platform (* $p < 0.05$, by ANOVA, n.s.- not significant).

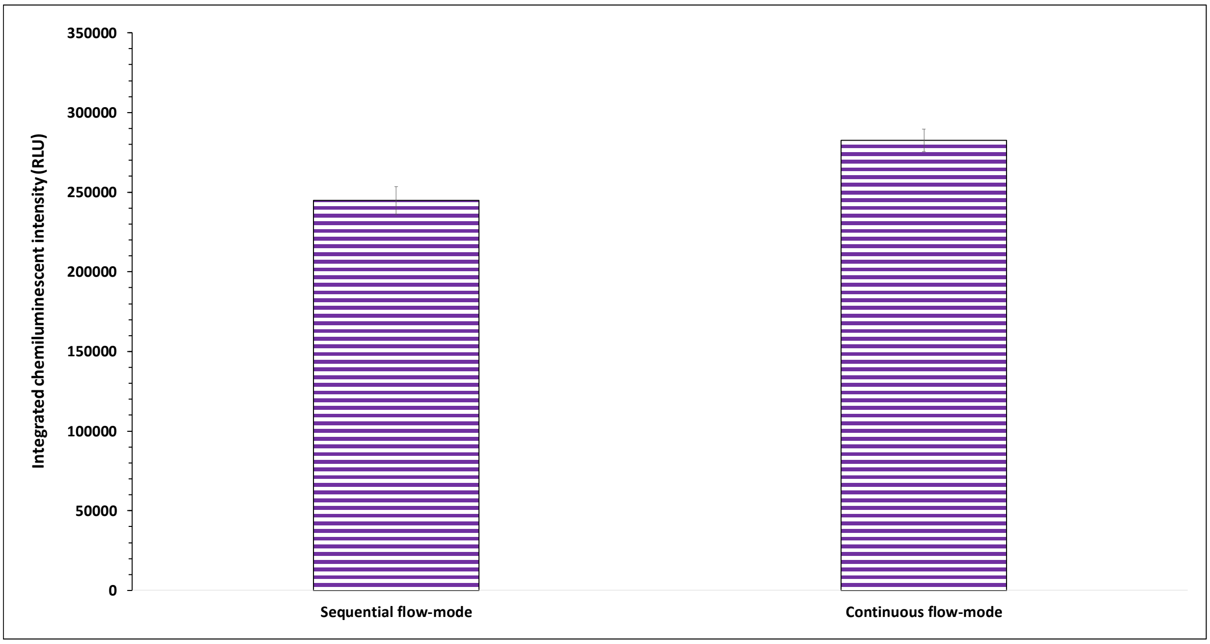

Figure S4. Effect of different types of sample addition modes on the integrated chemiluminescent intensity of VFIA platform.
